# Compliance to a Five-Year Biannual Ivermectin Treatment for Onchocerciasis Elimination and Its Determinants among Adults in the Bench Maji Zone, Southwest Ethiopia: A Community-Based Cross-Sectional Study

**DOI:** 10.1155/2021/8866639

**Published:** 2021-03-29

**Authors:** Bedilu Kifle, Mamo Nigatu

**Affiliations:** ^1^Bench Maji Zonal Health Department, Ethiopia; ^2^Jimma University, Institute of Health, Public Health Faculty, Epidemiology Department, Ethiopia

## Abstract

**Background:**

Community-directed treatment with ivermectin twice a year is a major action to control onchocerciasis in endemic countries. Even though the community-directed treatment with ivermectin was proven effective in treating the disease, the level of compliance to the treatment and its contributing factors was not well addressed in our study area. Therefore, the current study was aimed at determining the magnitude of compliance with the five-year (2013-2017 years) biannual ivermectin treatment and its associated factors among adults living in the Bench Maji Zone, Ethiopia.

**Methods:**

A community-based cross-sectional study was done on 572 randomly selected people aged greater than or equal to 15 years. Data were collected by a face-to-face interview. Descriptive statistics were used to summarize descriptive data. Binary logistic regression was done to assess statistical associations. Adjusted odds ratio and its 95% CI were, respectively, used to measure the strength of statistical association and its significance.

**Result:**

Five hundred fifty-three (553) people had participated in the study making the response rate 96.7%. The overall magnitude of compliance to the five-year biannual ivermectin treatment was 361 (65.3%). The results of the multivariable logistic regression showed that age, positive attitude towards community drug distributers' performance, positive attitude towards height measurement for the treatment dose determination, and involvement in community-directed treatment with ivermectin were independently associated with compliance to ivermectin treatment at *P* value < 0.05.

**Conclusions:**

Even though the Ethiopian government has set a goal to eliminate onchocerciasis through community-directed treatment with the ivermectin, which is proven effective in treating the disease, the magnitude of compliance with the treatment among adults aged ≥15 years in the Semen Bench District is still unacceptably low. The Bench Maji Zonal Health Department and other stakeholders working on onchocerciasis prevention, control, and elimination should give due emphasis to behavioral change communication through community-based education and other social media to promote community's awareness on community-directed treatment with ivermectin giving due focus to adults aged 45 years and above.

## 1. Background

Onchocerciasis, also known as river blindness, is a parasitic disease caused by a filarial worm called *Onchocerca volvulus*. Person-to-person transmission of the disease is due to the blood-feeding black fly of the genus *Simuliidae* [1, 2]. Infection with this nematode filarial worm leads to skin disease and anatomical impairment, which are dermatitis, pruritus or itching, depigmentation of the skin or leopard skin, onchocercomata, hanging groin, and temporary vision loss to blindness [2, 3]. People who live in fertile land and extensive agricultural farming area and those who live near river banks are more vulnerable to onchocerciasis infection [4–6].

According to the 2019 WHO estimate, there were 20.9 million *O. volvulus* infections worldwide of whom 14.6 million people had a skin disease and 1.15 million had vision loss [2]. On the other hand, the 2020 WHO African region report depicted that over 18 million people were infected by *O. volvulus* [1]. Onchocerciasis is endemic in many tropical countries but mainly in the equatorial region of Africa. The 2019 and the 2020 WHO report, respectively, showed that more than 99% and more than 80% of the infected cases live in 31 African countries [1, 2]. Different studies done in Ethiopia showed that the disease is still more prevalent in western and southwestern parts of the country with the prevalence range of 6.32% in the Bench Maji Zone to 31.3% in indigenous communities of Assosa [7–9].

Even though Africa is reaching the last milestone in eliminating the disease, there are still myriads of challenges impeding the elimination program among which incomplete elimination mapping of all transmission zones, coendemicity of onchocerciasis and loiasis, the possible emergence of ivermectin resistance, uncoordinated cross-border elimination efforts, conflict, and civil unrest, suboptimal program implementation, and technical and financial challenges are the majors [10]. Ethiopia launched an onchocerciasis elimination program in 2013 to attain interruption of onchocerciasis transmission nationwide by 2020. However, the transmission of the disease remained unabated in many districts despite many years of ivermectin MDA with the treatment coverage of more than 80% [6].

Community-directed treatment with ivermectin (CDTI) twice a year is a major action to control onchocerciasis in endemic countries which minimizes microfilaria load and cease reproductive life span of adult worm within 15-20 years of treatment [11]. Treatment with ivermectin is safe for any person aged 5 years and above, except for pregnant women, breastfeeding mothers for less than one week baby, and seriously ill persons [12].

Even though the Federal Ministry of Health of Ethiopia had set a goal to eliminate onchocerciasis and to stop community-directed treatment with ivermectin by the end of 2020, it was shifted to 2025 due to the potential transmission of the disease in the endemic areas [13]. In our study area, the Bench Maji Zone, the annual and the biannual community-directed treatment with ivermectin (CDTI) to control and eliminate the disease were started in 2003 and 2013, respectively; however, the prevalence study done in the area showed that the disease prevalence was still 6.32% [8]. Even though the aforementioned community-directed treatment with ivermectin (CDTI) was proven effective in treating the disease, the level of compliance to the treatment was not well addressed in our study area. Therefore, the current study was aimed at determining the level of compliance to the five-year (2013-2017 years) biannual ivermectin treatment and at identifying its predictors among adults aged ≥15 years living in Bench Maji Zone, Ethiopia.

## 2. Methods

### 2.1. Study Area and Period

**Figure 1:**
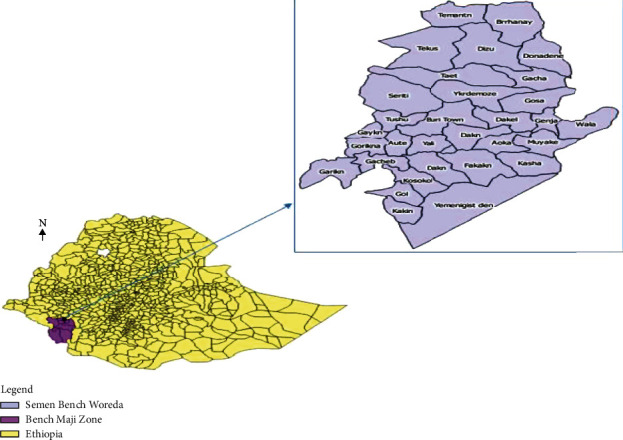
Map of Semen Bench District.

This study was conducted in Semen Bench District, Bench Maji Zone, Southwest Ethiopia, which is 568 km far from Addis Ababa, the capital city of the country, from April 1 to 28, 2018. Semen Bench District is one of the ten decentralized districts in Bench Maji Zone and 17 km far from the zonal administration town of Mizan Aman (Figure 1). The district has an altitude of 2000 m above sea level. The majority of the area is covered by broad-leafed rainforest and a predominant coffee growing area. There are many rivers and streams used for agriculture and home consumption. The livelihoods of the residents mainly depend on cultivating crops and breeding livestock. An administrative division of Semen Bench Districts comprised of 28 rural and 3 urban kebeles which is the lowest administrative division of Ethiopia. The total 2017/18 projected population size of the district is 138,556; from these male accounts, 67,892 and the rest 70,664 were females. There are 28,277 estimated households. More than 85% of the population lives in rural areas. There are four health centers and 31 health posts in the district. Community-directed treatment with ivermectin for onchocerciasis elimination had been going in the district annually for the last ten years and biannually for the last 5 years by mass drug administration with varying geographical coverage between 95 and 100% and therapeutic coverage above 80%.

### 2.2. Study Design and Population

A community-based cross-sectional study was conducted. Selected adults who had stayed in the Semen Bench District for five years and above, and who had been recorded since 2013, at the start of the biannual ivermectin treatment, had participated in the study. Study participants who had taken all the five-year (2013-2017) biannual ivermectin treatments or the ten treatment rounds were categorized as compliant to the treatment.

### 2.3. Sample Size and Sampling Techniques

The sample size was calculated by single population proportion formula by assuming 80.8% prevalence of compliance with ivermectin treatment from the study done at Southwest Ethiopia [14], 95% CI, 5% margin of error, 20% nonresponse rate, and a design effect of 2. The final sample size was 572 people. There are 3 urban and 28 rural kebeles (the lowest administrative unit) in the Semen Bench District. Nine rural kebeles and one urban kebeles were randomly included in the study. The calculated sample size (572) was allocated by probability proportional to size (PPS) to the selected kebeles based on the number of households in each kebele. Households were selected by systematic random sampling technique to obtain study participants from households. Only one study participant per household was randomly included in the study. In a case when there were two or more eligible individuals per household, one eligible individual was randomly selected by the lottery method.

### 2.4. Data Collection Techniques and Measurements

The independent variables included in this study include age, sex, educational status, ethnicity, religion, occupation, duration of stay in the residency area, marital status, the perceived performance of CDDs, family support, the perception that measuring height is good for dose determination, and involvement in CDTI. Data were collected by face-to-face interview using a structured questionnaire adapted from different works of literature [11–17]. CDTI village treatment registers used to identify compliance with ivermectin treatment of study participants were obtained from HEW who served as community health workers at the health posts. CDTI village register was printed and issued by FMoH and partners and was used to record all family members in the household and to record for eligible individuals' treatment status on every treatment round for five years.

Eight public health workers who were fluent in the local language were assigned for data collection. Two public health officers were also assigned to oversee the overall data collection process. Two health extension workers (HEWs) were assigned in each kebele to guide the data collectors. A two-day training was given for data collectors and supervisors before actual data collection. Before the commencement of actual data collection, pretesting of the tools was done in another district on 29 people with similar characteristics. Data collection tools were refined based on the results from the pretest.

### 2.5. Data Processing and Analysis

Data were entered using EpiData version 3.1 and exported to SPSS version 20 for statistical analysis. Descriptive statistics were done to summarize descriptive data. Bivariate logistic regression was performed to identify candidate variables for multivariable logistic regression. Candidate variables with a *P* value < 0.25 cutoff point in the bivariate analysis were entered into a multivariable logistic regression to identify independent predictors of compliance with ivermectin treatment and to control confounders. Adjusted odds ratio with its 95% CI was calculated to measure the strength of associations and its statistical significance, respectively. 95% CI was used to declare statistical significance in the final model.

### 2.6. Ethical Considerations

An ethical approval letter was obtained from the Ethical Review Board of the Institute of Health, Jimma University. Permission to conduct the study was obtained from the Bench Maji Zonal Health Department and Semen Bench District Health Office.

## 3. Result

### 3.1. Sociodemographic Characteristics of Study Participants

Five hundred fifty-three (553) people participated in the study making the response rate 96.7%. Almost half, 49.1% of the study subjects were male participants and thirty-eight percent were found between 15 and 24 years of age. 94.3 percent of the study participants were Bench in ethnicity, and nearly ten percent were protestant Christians. Regarding their educational status, nearly one-third (33.6%) had not attended any formal education. The majority of the study participants (63.7%) were farmers. 62.4% had stayed in the study area for more than 21 years and above (Table 1).

### 3.2. Prevalence of Compliance to Ivermectin Treatment

The current study revealed that only 361 (65.3%) study participants had fully complied with the five-year biannual or the ten-round treatments of ivermectin for onchocerciasis elimination in the study area. From the studied kebeles, the highest compliance to ivermectin treatment (70%) was detected in the kaiken kebele, while the lowest rate (53.7%) was detected in the Yeker Demos kebele (Table 2). The study also revealed that the highest (82.2%) and the lowest (53.6%) compliance to the ivermectin treatment were, respectively, detected in the age group of 35-44 years and ≥45 years. Nearly similar, 64% males and 66.5% females, respectively, complied with the treatment. Only 55.6% of farmers had complied with the treatment, while it was 82.5% among the other occupations (Table 1). More than three-fourths (75.7%) of study participants who had been involved in the CDTI complied with the treatment, while only 5.2% of those who had not been involved in CDTI complied with the treatment. 67.4% of study participants who had a positive attitude towards height measurement for dose determination complied with the treatment, while only 48.4% of the participants who had not the positive attitude towards the height measurement complied with the treatment. More than two-thirds (69%) of the study participants who had a positive attitude towards community drug distributors' performances complied with the treatment; whereas, only 55.6% of those who had not the positive attitude towards community drug distributors' performances complied with the treatment (Table 3).

### 3.3. Behavioral and Individual Characteristics of the Study Participants

Nearly two-thirds (66%) of the study participants had perceived the risk of acquiring the disease. More than three-fourths (77.8%) had family support, and more than two-thirds (72.6%) had a positive attitude towards community drug distributors' performance. 88.8% of the study participants had a positive attitude towards height measurement for dose determination of the treatment. More than nine in ten (91.6%) had known at least one CDD, and more than half of them (58.9%) were involved in CDTI (Table 3).

### 3.4. Result of the Bivariate Analysis

Bivariate logistic regression was done to select candidate variables for multivariable logistic regression analysis. Accordingly, age, marital status, positive attitude towards CDDS' performance, family support, positive attitude towards height measurement for treatment dose determination, and involvement in CDTI were significantly associated (*P* value < 0.25) with compliance with the ivermectin treatment and selected as candidate variables for multivariable logistic regression analysis (Table 4).

### 3.5. Independent Predictors of Compliance to Ivermectin Treatment

To identify independent predictors of compliance with ivermectin treatment, multivariable logistic regression was performed. Accordingly, age, positive attitude towards CDDS' performance, positive attitude towards height measurement for treatment dose determination, and involvement in CDTI were independently associated with compliance to ivermectin treatment at *P* value < 0.05.

The finding of this study showed that age was independently associated with compliance to ivermectin treatment. An adult person in the age group of 25-34 years was 3.2 times more likely to comply with ivermectin treatment as compared to an adult in the age group of 45 and above years of age (AOR = 3.260, 95% CI: 1.363, 4.831). Similarly, an adult whose age was between 35 and 44 years was nearly four times more likely compliant with ivermectin treatment as compared to an adult who was in the age group of 45 and above years of age (AOR = 3.645, 95% CI: 1.313, 4.962).

The study also revealed that a positive attitude towards the community drug distributors' performance was independently associated with compliance to ivermectin treatment. Study participant who had a positive attitude towards community drug distributors' performance was two times more likely to comply with ivermectin treatment as compared to study participant who did not have positive attitude (AOR = 2.02, 95% CI: 1.42, 5.92).

The study also revealed that compliance with ivermectin treatment was independently predicted by a positive attitude towards height measurement for treatment dose determination. People who had a positive attitude towards height measurement for treatment dose determination were more than two times more likely to be compliant with ivermectin treatment as compared to their counterparts (AOR = 2.304, 95% CI: 1.624, 3.485).

This study also depicted that involvement in CDTI was an independent predictor of compliance with ivermectin treatment. People who were involved in CDTI were nearly three times more likely to be compliant with ivermectin treatment as compared to people who did not involve in CDTI (AOR = 2.956, 95% CI: 1.393, 6.694) (Table 5).

## 4. Discussion

This study was aimed at determining the prevalence of compliance with the five-year (2013-2017) biannual ivermectin treatment and at identifying its predictors among adults aged ≥15 years in Bench Maji Zone, to shed a light on the progress of onchocerciasis elimination. The study revealed that the overall prevalence of compliance with the five-years biannual ivermectin treatment in the Semen Bench District was 65.3% which is comparable with the separate study done in the west region of Cameroon and the three other regions of the same country where 67.1% and 64.1% of study participants had, respectively, complied to ivermectin treatment [15, 16]. But the report from the study done in Southwest Ethiopia showed that 80.8% of the study participants were compliant to ivermectin treatment [17] which is higher than the level of compliance from the current study. The discrepancy may be explained by differences of awareness of the communities on the disease and the community-directed treatment with ivermectin (CDTI). Similarly, the current result is also inconsistent with the finding of the study from Cameroon and Nigeria where the compliance rate of ivermectin treatment was lower (42.9%) [14]. The difference might be explained by differences in access to CDTI and differences in programmatic management of CDTI across different regions of Africa.

The current study also showed that age was independently associated with compliance to the ivermectin treatment. An adult in the age group of 25-34 years was more than 3 times more likely to comply with the ivermectin treatment as compared to an adult in the age group of 45 and above (AOR = 3.260, 95% CI: 1.363, 4.831). Similarly, an adult whose age was between 35 and 44 years was nearly four times more likely compliant with the ivermectin treatment as compared to an adult who was in the age group of 45 and above (AOR = 3.645, 95% CI: 1.313, 4.962). The finding of this study was in line with the study conducted on onchocerciasis control in the Cabo area, Southwest Ethiopia, Cameroon, and Nigeria [14, 17]. This could be explained by different reasons. In the context of our population, older people are, most of the time, the breadwinner of their families and are more engaged in fieldwork than young people and maybe absent from home during the drug allocations.

The study showed that a positive attitude towards the CDDS' performance was independently associated with compliance with ivermectin treatment. The study participant who had a positive attitude towards the CDDS' performance was two times more likely to comply with the ivermectin treatment as compared to the study participant who did not have a positive attitude towards the CDDS' performance (AOR = 2.021, 95% CI: 1.422, 5.921). The finding of this study is congruent with studies done in Southwest Ethiopia and Cameroon [14, 17]. This might be due to a lack of trust in the performance of CDDS.

The study also revealed that compliance with ivermectin treatment was independently predicted by a positive attitude towards height measurement for the treatment dose determination. Study participants who had a positive attitude towards height measurement for the treatment dose determination were more than two times more likely to comply with the ivermectin treatment as compared to their counterparts (AOR = 2.304, 95% CI: 1.624, 3.485). The finding of this study is also consistent with the study done on factors associated with compliance to ivermectin treatment for onchocerciasis control in Bebeka coffee plantation, Southwest Ethiopia [17].

In the current study, involvement in the CDTI was independently associated with compliance with the ivermectin treatment. People who were involved in the CDTI were nearly three times more likely compliant with the ivermectin treatment as compared to people who did not involve in the CDTI (AOR = 2.956, 95% CI: 1.393, 6.694). The finding of this study was also in agreement with the study conducted on the audit of community-directed treatment with ivermectin for onchocerciasis and factors associated with adherence in the three regions of Cameroon [16]. This could be due to the reason that peoples who had been involved in community-directed treatment with ivermectin had more awareness on the community drug distribution, knew the place where the treatment is given, and knew the schedules when the treatment is given in their villages than their counterparts who had not been involved in the community-directed treatment with ivermectin.

This study has its limitations. Since secondary data were extracted from registrations, the accuracy of the information is up to the original registration and could not be ascertained. On the other hand, the temporality between adherence to the ivermectin treatment and the associated factors could not be ascertained due to the nature of the study design and the evidence should be used with caution. Furthermore, we were also not able to assess the effects of sociocultural factors on compliance to the ivermectin treatment due to a lack of qualitative data from the registrations.

## 5. Conclusions

Even though the Ethiopian government has set a goal to eliminate onchocerciasis through community-directed treatment with the ivermectin treatment, which is proven effective in treating the disease, the magnitude of compliance with the treatment among adults aged ≥15 years in the Semen Bench District is still unacceptably low. Age, positive attitude towards CDDS' performance, positive attitude towards height measurement for the treatment dose determination, and involvement in CDTI were independently associated with the compliance with the ivermectin treatment. Therefore, the Bench Maji Zonal Health Department and other stakeholders working on onchocerciasis prevention, control, and elimination should give due emphasis to behavioral change communication through community-based education and other social media to promote community's awareness on CDTI giving due focus to adults aged 45 years and above. Involvement in the CDTI should also be encouraged by the stakeholders.

## Figures and Tables

**Table 1:** Sociodemographic characteristics of study participants in Semen Bench District, Bench Maji Zone, Southwest Ethiopia, June 2018 (*n* = 553).

Variable	Category	Total (%)	Compliance status	
			Compliant	Noncompliant
Age	15-24	210 (38)	106 (49.5)	104 (50.5)
	25-34	171 (30.9)	137 (80.1)	34 (19.9)
	35-44	90 (16.3)	74 (82.2)	16 (17.8)
	≥45	82 (14.8)	44 (53.6)	38 (46.4)

Sex	Male	272 (49.1)	174 (64)	98 (36)
	Female	281 (50.9)	187 (66.5)	94 (33.5)

Ethnicity	Bench	522 (94.3)	342 (65.6)	180 (35.4)
	Others	31 (5.7)	19 (61.3)	12 (38.7)

Marital status	Married	189 (34.1)	137 (72.4)	52 (27.6)
	Others	364 (65.9)	224 (61.5)	140 (38.5)

Religion	Protestant	496 (89.6)	328 (66.1)	168 (33.9)
	Others	57 (10.4)	33 (57.8)	24 (42.2)

Educational status	Illiterate	186 (33.6)	87 (46.8)	99 (53.2)
	Literate	367 (66.4)	274 (74.6)	93 (25.4)

Occupation	Farmer	352 (63.7)	195 (55.4)	157 (44.6)
	Others	201 (36.3)	166 (82.5)	35 (17.5)

Duration of stay in years	5-20	208 (37.6)	105 (50.4)	103 (49.6)
	21 and above	345 (62.4)	256 (74.2)	89 (25.8)

**Table 2:** Prevalence of compliance to ivermectin treatment among the studied kebeles in the Semen Bench District, Bench Maji Zone, Southwest Ethiopia, June 2018(*n* = 553).

Kebele	Number of participants	Compliant	Noncompliant	Prevalence (%)
Kaiken	40	28	12	70
Gazyken	36	24	12	66.7
G/mag	77	43	34	55.8
Wesheken	76	52	24	68.4
Gacheb	15	9	6	60.0
Eusken	69	41	28	59.4
Y/demos	67	36	31	53.7
Kosokol	40	29	11	72.5
Gariken	63	52	11	82.5
Kasha	70	47	23	67.1
Total	553	361	192	65.3

**Table 3:** Behavioral and individual characteristics of the study participants in the Semen Bench District, Bench Maji Zone, Southwest Ethiopia, June 2018 (*n* = 553).

Variables	Category	Total (%)	Compliance status	
			Compliant (%)	Noncompliant (%)
Perceived risk of acquiring the disease	Yes	488 (88.2)	322 (66)	166 (34)
	No	65 (11.8)	39 (60)	26 (40)

Positive attitude towards community drug distributers' performance in allocating the treatment	Yes	402 (72.6)	277 (69)	118 (31)
	No	151 (27.4)	84 (55.6)	74 (54.4)

Family support	Yes	430 (77.8)	292 (67.9)	138 (31.9)
	No	123 (22.2)	69 (56.1)	54 (43.9)

Positive attitude towards height measurement for dose determination	Yes	491 (88.8)	331 (67.4)	160 (32.6)
	No	62 (11.2)	30 (48.4)	32 (51.6)

Know at least one CDD	Yes	507 (91.6)	334 (65.8)	173 (34.2)
	No	46 (8.4)	27 (58.7)	19 (41.3)

Involvement in CDTI	Yes	326 (58.9)	247 (75.7)	79 (24.3)
	No	227 (41.1)	114 (5.2)	113 (49.8)

**Table 4:** Results of the bivariate analysis of predictors of compliance with ivermectin treatment in the Semen Bench District, Bench Maji Zone, Southwest Ethiopia, June 2018 (*n* = 553).

Variable	Category	Total (%)	Compliance status		COR (95% CI)	*P* value
			Compliant	Noncompliant		
Age	15-24	210 (38)	106 (49.5)	104 (50.5)	0.880 (0.874, 1.966)	0.325
	25-34	171 (30.9)	137 (80.1)	34 (19.9)	3.359 (1.253, 4.632)	0.001^∗^
	35-44	90 (16.3)	74 (82.2)	16 (17.8)	3.994 (1.992, 5.228)	<0.001^∗^
	≥45	82 (14.8)	44 (53.6)	38 (46.4)	1.000	

Sex	Male	272 (49.1)	174 (64)	98 (36)	0.892 (0.466, 1.221)	0.661
	Female	281 (50.9)	187 (66.5)	94 (33.5)	1.000	

Ethnicity	Bench	522 (94.3)	342 (65.6)	180 (35.4)	1.200 (0.557, 2.559)	0.553
	Others	31 (5.7)	19 (61.3)	12 (38.7)	1.000	

Marital status	Married	189 (34.1)	137 (72.4)	52 (27.6)	1.647 (0.968, 1.963)	0.075^∗^
	Others	364 (65.9)	224 (61.5)	140 (38.5)	1.000	

Religion	Protestant	496 (89.6)	328 (66.1)	168 (33.9)	2.037 (0.852, 4.663)	0.331
	Others	57 (10.4)	33 (57.8)	24 (42.2)	1.000	

Educational status	Illiterate	186 (33.6)	87 (46.8)	99 (53.2)	1.000	
	Literate	367 (66.4)	274 (74.6)	93 (25.4)	0.298 (0.442, 1.339)	0.454

Occupation	Farmer	352 (63.7)	195 (55.4)	157 (44.6)	1.057 (0.847, 1.696)	0.524
	Others	201 (36.3)	166 (82.5)	35 (17.5)	1.000	

Duration of stay in years	5-20	208 (37.6)	105 (50.4)	103 (49.6)	1.000	
	21 and above	345 (62.4)	256 (74.2)	89 (25.8)	0.354 (0.377, 2.669)	0.443

Perceived risk of acquiring the disease	Yes	488 (88.2)	322 (66)	166 (34)	1.293 (0.320, 4.221)	0.668
	No	65 (11.8)	39 (60)	26 (40)	1.000	

Positive attitude towards CDDS' performance	Yes	402 (72.6)	277 (69)	118 (31)	2.068 (0.588, 6.723)	0.187^∗^
	No	151 (27.4)	84 (55.6)	74 (54.4)	1.000	

Family support	Yes	430 (77.8)	292 (67.9)	138 (31.9)	1.656 (0.968, 2.717)	0.233^∗^
	No	123 (22.2)	69 (56.1)	54 (43.9)	1.000	

Positive attitude towards height measurement for treatment dose determination	Yes	491 (88.8)	331 (67.4)	160 (32.6)	2.207 (1.516, 3.484)	0.009^∗^
	No	62 (11.2)	30 (48.4)	32 (51.6)	1.000	

Know at least one CDD	Yes	507 (91.6)	334 (65.8)	173 (34.2)	1.359 (1.541, 4.442)	0.476
	No	46 (8.4)	27 (58.7)	19 (41.3)	1.000	

Involved in CDTI	Yes	326 (58.9)	247 (75.7)	79 (24.3)	3.099 (1.770, 9.516)	0.024^∗^
	No	227 (41.1)	114 (5.2)	113 (49.8)	1.000	

^1^Reference. ^∗^Significant variable.

**Table 5:** Independent predictors of compliance to ivermectin treatment in the Semen Bench District, Bench Maji Zone, Southwest Ethiopia, June 2018(*n* = 553).

Variables	Categories	Compliant (*n* = 361) (65.2%)	Noncompliant (*n* = 192) (34.8%)	COR (95% CI)	AOR (95% CI)
Age in year	15-24	106 (49.5)	104 (50.5)	0.880 (0.874, 1.966)	0.68 (0.77, 1.76)
	25-34	137 (80.1)	34 (19.9) 16 (17.8)	3.36 (1.25, 4.63)	3.26 (1.37, 4.83)^∗^
	35-44	74 (82.2)	38 (46.4)	3.99 (1.99, 5.23)	3.645 (1.31, 4.96)^∗^
	45 and above	54 (53.6)		1.000	1

Positive attitude towards CDDS' performance	Yes	277 (69)	118 (31)	2.07 (0.59, 6.72)	2.021 (1.42, 5.9)^∗^
	No	84 (55.6)	74 (54.4)	1.000	1.000

Positive attitude towards height measurement for treatment dose determination	Yes	331 (67.4)	160 (32.6) 32 (51.6)	2.207 (1.52, 3.48)	2.30 (1.62, 3.49)^∗^
	No	30 (48.4)		1.000	1.000

Involved in CDTI	Yes	247 (75.7)	79 (24.3)	3.10 (1.77, 9.52)	2.96 (1.39, 6.70)^∗^
	No	114 (5.2)	113 (49.8)	1.000	1.000

^1^Reference. ^∗^Significant variable.

## Data Availability

The data sets used and/or analyzed during the current study are available from the corresponding author on reasonable request.

## Abbreviations

AOR: Adjusted odds ratio
CDD: Community drug distributors
CDTI: Community-directed treatment with ivermectin
CI: Confidence interval
FMoH: Federal Ministry of Health
HEW: Health extension worker
km: Kilometer
MDA: Mass drug administration
Mf: Microfilaria
NTD: Neglected tropical disease
PPS: Probability proportional to size
SPSS: Statistical Package for the Social Sciences
WHO: World Health Organization.
